# Adrenal Gland Necrosis in Pregnancy: How to Manage? Case Series at Nantes University Hospital and Literature Review

**DOI:** 10.3390/jcm12186036

**Published:** 2023-09-18

**Authors:** Mathilde Guyon, Manon Degez, Mathieu Artifoni, Thomas Goronflot, Emilie Misbert, Vincent Dochez, Norbert Winer

**Affiliations:** 1 Department of Gynecology-Obstetrics, Nantes University Hospital, 44000 Nantes, Francevincent.dochez@chu-nantes.fr (V.D.); norbert.winer@chu-nantes.fr (N.W.); 2 Department of Internal Medicine, Nantes University Hospital, 44000 Nantes, France; mathieu.artifoni@chu-nantes.fr; 3 Data Clinic, National Institute for Health and Medical Research, Nantes University Hospital, 44000 Nantes, France; thomas.goronflot@chu-nantes.fr

**Keywords:** adrenal necrosis, adrenal ischemia, adrenal thrombosis, nonhemorrhagic adrenal infarction, abdominal pain in pregnancy, adrenal insufficiency

## Abstract

Adrenal necrosis is a rare but serious cause of abdominal pain of thrombotic origin during pregnancy. There is often a delay in diagnosis and treatment. The objective was to specify the clinical and paraclinical signs suggestive of adrenal necrosis, making it possible to improve the delay in diagnostic. The secondary objective was to establish a multidisciplinary protocol regarding management. This is a case report of pregnant women with a radiological diagnosis of adrenal gland necrosis. In parallel, we carried out a systematic review in the same period. We studied these patients’ clinical, biological and radiological data. We included eight patients with a computed tomography scan diagnosis of adrenal necrosis and fifteen articles in the literature describing twenty-four cases. All the patients presented with the same symptoms. The treatment was based on curative anticoagulation. The diagnosis of adrenal gland necrosis is worth suggesting in view of the array of sudden morphine-resistant abdominal pain associated with a biological inflammatory syndrome. The diagnosis is based on the computed tomography scan. Three to six months of curative anticoagulation is recommended as well as a thrombophilia and endocrinological assessment to rule out adrenal insufficiency.

## 1. Introduction

Adrenal gland necrosis is described as a rare but serious cause of abdominal pain during pregnancy, mainly during the third trimester with few cases described in the literature. According to Glomksi et al. [1], the incidence was 1.3% according to their ten-year study of magnetic resonance imaging (MRI) for acute abdominal pain at Brigham and Women’s Hospital in Boston. The severity of the pathology is linked to a possible adrenal insufficiency in the event of bilateral involvement. Clinical signs are nonspecific and can mimic other more common pathologies [2] (such as appendicitis, pulmonary embolism), biliary pathology (such as hepatic colic or cholecystitis), urinary pathology (such as renal colic or pyelonephritis) or even gynecological pathologies (such as adnexal torsion or hemolysis, elevated liver enzymes and low platelet count syndrome (HELLP)). The most frequent signs are sudden and intense abdominal pain, mainly in the right hypochondrium, associated with nausea and vomiting. Biological signs are also nonspecific with an isolated inflammatory syndrome and do not, alone, allow the diagnosis to be made. Faced with this nonspecific clinical-biological picture, there is often a delay in diagnosis and treatment.

As an anatomical reminder [3], adrenal glands are retroperitoneal and nonsymmetrical endocrine glands overhanging the right and left kidneys. They secrete catecholamines, androgen hormones, mineralocorticoids and glucocorticoids. Arterial vascularization is based on a rich network made up of three arteries: superior, middle and inferior, on the right and on the left. On the contrary, the venous system consists of a single adrenal vein, the true secretory duct of the gland. On the right, the adrenal vein empties into the inferior vena cava, while the left adrenal vein enters the left renal vein, which itself enters the inferior vena cava. Various terms are frequently used (adrenal hemorrhagic necrosis, adrenal hemorrhagic infarction, spontaneous adrenal hematoma), but adrenal hematoma is most often the result of adrenal vein thrombosis [4].

Several cases were diagnosed at the Nantes University Hospital Center during the year 2022. Faced with the discrepancy between a pathology described as rare and the frequency of cases in our hospital, we were interested in the cases that occurred in our Center over the past ten years, between 2012 and 2022.

## 2. Materials and Methods

This is a case report of 8 patients with adrenal necrosis during pregnancy at the Nantes University Hospital, between January 2012 and October 2022. We started collecting the files using the CIM 10 codes relating to the pathology. As this method did not find any records, the cases studied were found by epidemiologists from the Nantes University Hospital Data Clinic, using the following keywords: “thrombosis”, “adrenal” and “pregnancy”. We therefore collected twelve cases, among which, four were excluded: postpartum context, thrombosis outside of pregnancy, fetal adrenal thrombosis and one diagnosis invalidated during the patient’s follow-up.

**Figure 1:**
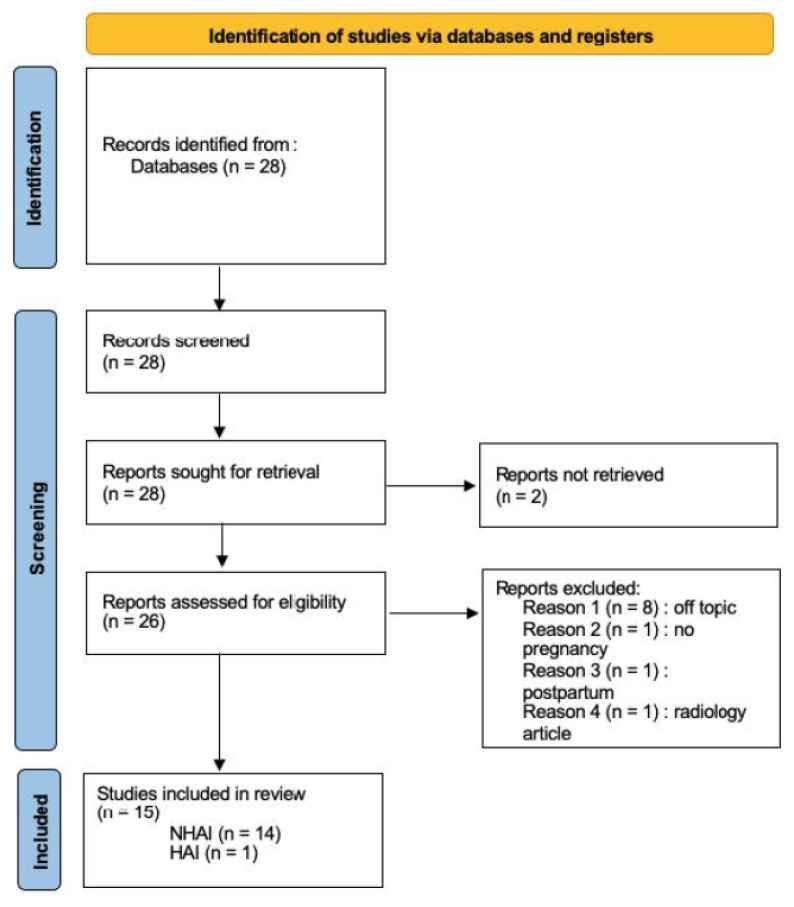
Prisma flowchart (NHAI: nonhemorrhagic adrenal infarction; HAI: hemorrhagic adrenal infarction).

Regarding the systematic review, we collected articles written over the same period from 2012 to 2022 via the PubMed search platform, using these keywords: “adrenal infarction” and “pregnancy”. Twenty-eight articles were found, of which thirteen were excluded (Figure 1). These were the reasons for exclusion: articles that were not accessible, off-topic, about other thrombosis and about pathologies outside of pregnancy. The remaining fifteen articles all reported cases enabling us to collect clinical, biological and radiological signs together with their respective management. Fourteen articles reported cases of nonhemorrhagic necrosis of the adrenal gland during pregnancy, and one article reported a case of adrenal gland hemorrhagic necrosis during pregnancy. Two authors participated to this screening.

## 3. Results

Eight cases were selected for our case report based on research carried out by epidemiologists at the Data Clinic at Nantes University Hospital. We included these eight patients, identified as A to H in Table 1, with a diagnosis of adrenal necrosis. All were diagnosed by a CT scan at the University Hospital.

Patients’ mean age was 27.2 years ((18–33), σ = 5.5) (Table 1). Their average body mass index (BMI) was 28.7 kg/m^2^ ((22.2–42), σ = 7.4). Three patients had notable antecedents, in particular thromboembolic. Patient D had a history of pulmonary embolism two days after taking estrogen–progestogen contraception, patient F had a kidney transplant for malformative uropathy with NODAT-type diabetes and a vaginal thrombus during her previous delivery by low way. Patient G had a type 1 Chiari malformation and a history of idiopathic reversible cerebral vasoconstriction syndrome. The other patients had no significant personal histories. Only patient A had reported consumption of tobacco and other toxic substances during pregnancy.

Obstetrically, pregnancies were unremarkable and involved singleton fetuses. The mean term at the time of diagnosis was 36 weeks of gestation ((32.5–40), σ = 2.4), ranging from 32 weeks for patient D to 39 SA weeks for patient G. The mean gestation was 2.25 ((1–4), σ = 1.3), and the mean parity was 0.63 ((0–3), σ = 0.7). Only two patients had spontaneous miscarriages in previous pregnancies. Patient C presented with threatened preterm labor (PMD) at 33 weeks, patient D was followed for gestational diabetes under diet and patient E for blood pressure without proteinuria.

Concerning the clinical picture, all the patients presented with similar symptoms with the exception of patient F, who presented with intense lumbar then bilateral dorsolumbar pain, without abdominal pain. The other patients presented with sudden, intense and transfixing abdominal pain in the right hypochondrium, morphine-requiring or even morphine-resistant accompanied by nausea and vomiting. All were apyretic. On admission, patient G presented with a blood pressure asymmetry, leading to immediate suspicion of aortic dissection. Several diagnoses were mentioned during admissions: lithiasis pathology, renal colic, preeclampsia with incomplete picture, thrombosis of the adrenal veins and aortic dissection. No patient reported any obstetric complaint.

Regarding the biological picture, it was also similar for all the patients. All presented with an isolated biological inflammatory syndrome. The rest of the assessments, namely liver assessment, lipase, ionogram, coagulation assessment, proteinuria, were systematically negative. Troponins, if measured, were also negative. None of the patients presented with adrenal insufficiency.

**Figure 2:**
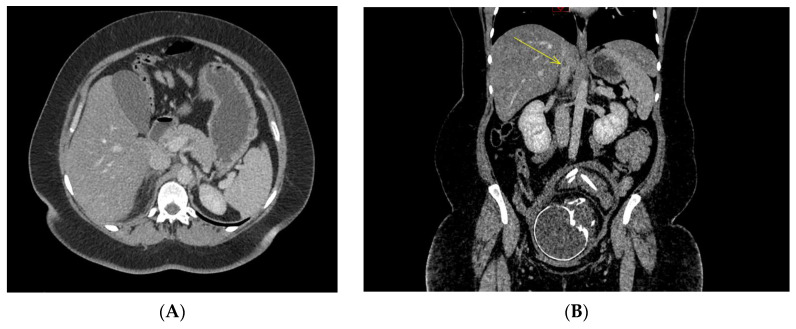
CT-scan sections ((**A**) infiltration of the right adrenal compartment with edematous appearance and overall hypo-enhancement of the right adrenal gland; (**B**) yellow arrow indicates venous thrombosis of the inferior vena cava complicated by hemorrhagic necrosis of the right adrenal gland).

Concerning the radiological picture, all patients had the same assessment, namely an obstetrical ultrasound by the intern and/or the senior on-call obstetrical emergency, an abdominal ultrasound and an abdominopelvic or thoraco-abdominopelvic CT scan. The obstetrical ultrasounds were all reassuring. Abdominal ultrasounds were normal or noncontributory for all cases. The computed tomography scans made it possible to diagnose adrenal glands necrosis. (Figure 2). There are seven necroses of the right adrenal gland in one case of bilateral involvement. There were six cases of nonhemorrhagic necrosis and two cases of adrenal gland hemorrhagic necrosis. Thrombosis, of the adrenal vein or the inferior vena cava, was not always visualized. The mean time to diagnosis at the Nantes University Hospital was 18 h.

Regarding the etiological assessment and management, each of the patients had a thrombophilia assessment during the diagnosis of adrenal necrosis with search for an antiphospholipid syndrome (lupus circulating anticoagulant = antiprothrombinase antibody, anticardiolipin antibody, antibodies toβ2Gp1), constitutional thrombophilia (assay of protein S, protein C, antithrombin and search for factor II and factor V mutations), myeloproliferative syndrome (JAK2 mutation) and paroxysmal nocturnal hemoglobinuria (PNH clone). The initial assessment of three patients (E, F, H) came back positive with a circulating lupus anticoagulant for each of the three. The thrombophilia assessment of the other five patients came back negative. In all patients, anticoagulant treatment was initiated as soon as the diagnosis was made. All received curative dose low molecular weight heparin. Patient F then received unfractionated heparin by electric syringe because it was more manageable in the event of an indication of birth in the context of immunosuppression in a renal transplant patient, and induction was scheduled for 37 weeks. Triggers for patients A and C were programmed at 39 weeks in order to organize a therapeutic window for anticoagulant treatment. In the cases of patients B and F with superimposed preeclampsia during pregnancy, maturation by prostaglandins then triggering started at 37 weeks. Patients D and H went into spontaneous labor at 37 weeks, before a therapeutic window and induction could be established. It was decided to promote spontaneous labor for patient E because of the contraindication to epidural analgesia. Due to suspicion of aortic dissection, patient G had a caesarean section on admission at 36 weeks. At birth, the newborns presented with no problems adapting. The pain quickly subsided after initiating anticoagulant treatment. All patients continued curative anticoagulant treatment for a minimum of 3 months or even 6 months after delivery. The relay treatments used were warfarin (*n* = 5) and rivaroxaban (*n* = 1).

Concerning remote follow-up, seven patients (B, C, D, E, F, G, H) had a CT scan three to four months later, showing remission of necrosis with sequela atrophy of the affected adrenal gland for the patients C, D, E and G. Compensatory hypertrophy of the contralateral gland was found in patient C. In patients B, F and H, the glands appeared normal. Patient A did not have follow-up imaging. A systematic control of the thrombophilia assessment at three months was also planned in view of the large number of false positives. The initially positive assessment with antiprothrombinase antibodies in patient E turned out to be negative at a distance and the antiprothrombinase antibodies in patient F remained positive at 6 months. At three months, an upper limit of Willebrand factor was found in patient H. It should be checked again at a later date.

We wanted to compare our population to the literature, by carrying out a systematic review of articles published over the same 2012 to 2022 period. That way, a systematic twenty-four case review, spread over fifteen articles [1,5,6,7,8,9,10,11,12,13,14,15,16,17,18], was made. Table 2 summarizes the clinical, biological, radiological and etiological presentations of these patients. All the articles found are case reviews, sometimes supplemented by reviews of the literature [8].

Concerning the clinical, biological and radiological pictures, we found similar presentations for all cases with acute, brutal and intense abdominal pain accompanied by an isolated biological inflammatory syndrome without obstetric complaint. None of the patients had a notable history. In 75% of cases, right adrenal gland involvement was found with nonhemorrhagic necrosis in more than 95% of cases. Only two items [5,6] reported adrenal insufficiency. In fourteen of the twenty-four cases, the thrombophilia assessment was negative.

Concerning therapeutic management, most articles reported the introduction of anticoagulant treatment, preventive or curative, for variable durations, ranging from the period of pregnancy up to 11 months postpartum. In five cases, the teams had not introduced anticoagulant treatment, in particular in the case of a patient who consulted for the first time for intense pain in the right hypochondrium at 17 weeks without finding etiology then the second time at 35 weeks for the recurrence of left lateral pain. The diagnosis of necrosis of the left adrenal gland had led to rereading the images of the first painful episode and leading to a posteriori conclusion of the right adrenal gland necrosis during the first consultation [16].

Control imaging, MRI and/or CT, were performed in eleven patients within variable delays, between one month and seven months from diagnosis. In the articles, we found results indicative of the normalization of the gland or of a sequela of atrophy with a possible contralateral gland hypertrophy.

By comparing the cases that occurred in our case report at the University Hospital Center of Nantes, with the cases found in the literature, we were able to establish several similarities (Table 3).

## 4. Discussion

This is an interesting review: it should raise awareness among obstetric teams of the diagnosis of adrenal thrombosis, which is probably still underdiagnosed.

In our descriptive analysis, we find similarities between our patients and the literature review [1,2,3,4,5,6,7,8,9,10,11,12,13,14,15]. Indeed, the epidemiological data of age, term, gestation and parity are similar. In terms of the attack laterality, it seems the right adrenal gland is more frequently affected than the left adrenal gland and on a nonhemorrhagic side in the majority of cases.

We therefore observed it was a pathology of the young woman, less than 30 years of age on average, future primiparous and without particular antecedent in the majority of the cases. Adrenal necrosis is the consequence of an atypical thrombosis and may be a diagnostic event of an underlying hemostasis disorder. However, these articles did not specify whether the thrombophilia assessments were rechecked remotely because of the risk of false positive antiprothrombinase antibodies taking part in the diagnosis of antiphospholipid syndrome. At the Nantes University Hospital, after yet another remote assessment, a patient retained positive antiprothrombinase antibodies, making the diagnosis of antiphospholipid syndrome (APS). In the other patients, no etiology other than pregnancy was found.

Pregnancy seemed to be the main contributing factor. Arnold et al. [19] published a series of autopsies providing information on the pathophysiology of this pathology. Whether the necrosis is hemorrhagic or not, it is based on a thrombotic phenomenon, mainly of the adrenal vein. To the anatomical and vascular specificities of the adrenal glands mentioned above are added the physiological pregnancy changes, such as the increase in blood volume, changes in the production of coagulation factors creating a state of hypercoagulability, as well as the mass effect of the gravid uterus increasing venous stasis. According to The American College of Obstetricians and Gynecologists (ACOG) from 2018 [20], pregnancy itself increases the risk of thromboembolism five times and the risk of venous thrombosis three to four times compared to a nonpregnant woman. Eighty percent of thromboembolic events during pregnancy are of venous origin [21,22].

Concerning endocrinological evaluation, it is important to differentiate serious and urgent glucocorticoid (cortisol) deficiency and mineralocorticoid (aldosterone) deficiency. Adrenal insufficiency is a serious and potentially lethal pathology [1] due to cortisol deficiency if not treated rapidly. This insufficiency was investigated by measuring cortisol and ACTH levels at 8 a.m. Collapsed cortisol levels and elevated ACTH indicate primary adrenal insufficiency due to damage to the adrenal gland. Adrenal insufficiency is not expected in the cases of unilateral involvement, since the contralateral gland continues to secrete adrenal hormones. Thus, the risk of adrenal failure in these patients remains theoretic, except in the exceedingly rare case of bilateral simultaneous adrenal infarction as reported by Songtanin et al. [5]. Moreover, no patients in our retrospective study presented with it, and only two cases have been described in the literature. The first one was reported by Shah et al. [5], concerning a unilateral adrenal necrosis. The authors do not explain the adrenal insufficiency despite it being a normal contralateral gland. Their patient received hydrocortisone supplementation and regular endocrinology follow-up with synacthene tests until normalization is achieved, allowing substitution to be discontinued. Shah et al. [6] reported a case of bilateral simultaneous adrenal infarction with intravenous hydrocortisone needs and switched after six days with oral supplementation adjusted by an ACTH stimulation test.

Concerning the imaging modalities, the contrast-enhanced CT is considered the gold standard imaging modality to rule out an adrenal ischemia. However, MRI is preferred over CT in pregnancies earlier than 25 weeks to protect of ionizing radiation and iodinated contrast material. Specific CT signs help to make the diagnosis without delay: spontaneous hypodensity and hypertrophy of the adrenal gland, slight enhancement after injection of contrast, periadrenal fatty infiltration, the “capsular sign” known as a hyperdense subtle peripheral line around a hypodense adrenal gland which is 100% specific of an early phase of adrenal ischemia and, less frequently, thrombosis of the adrenal vein; thrombosis might be extended into the lower vena cava and can be associated with iliac and pelvic veins thrombosis [23]. MRI features with morphologic abnormalities included unilateral adrenal enlargement, increased T2 signal intensity of the infarcted land with surrounding retroperitoneal edema and without T1 signal intensity suggesting hemorrhage [1].

Concerning therapeutic management, despite pain resistant to three-level analgesics, the pain seems to be relieved quickly when anticoagulant treatment is initiated. The benefit of this treatment is, in addition to analgesia, to prevent the progression of the thrombosis and the appearance of contralateral involvement. In the cases in our review, the duration of anticoagulation was variable. According to the ACOG [24], a 3 to 6 month curative anticoagulation is recommended in the cases of thrombosis. Nevertheless, after the initial treatment, it is advisable to continue curative or preventive anticoagulation for the rest of the pregnancy and up to six weeks postpartum. However, there are no French recommendations specifically concerning adrenal vein thrombosis during pregnancy. Pregnancy should not be an obstacle to curative anticoagulation and should be managed with the organization of a peripartum therapeutic window, jointly with the anesthesiologists.

Concerning the follow-up to be organized, both biologically and radiologically, the rarity of the pathology does not make it possible to establish a consensus. However, imaging such as computed tomography or MRI seems to be indicated to make the diagnosis. Remote imaging is most often performed within a few months after diagnosis to look for hemorrhagic transformation during revascularization, sequelae atrophy of the gland or normalization of the affected gland. The benefit of anticoagulation during a future pregnancy is discussed, especially in the case of associated risk factors. However, it is not recommended systematically by The American College of Obstetrics and Gynecologists in view of results inconsistency and the lack of effects found in the existing meta-analyses [24,25]. Further studies are needed to establish groups of patients in whom anticoagulation could be systematically recommended.

Patients with this history should be discussed at a multidisciplinary meeting with internists and hemostasis specialists for the management and monitoring of a new pregnancy.

We can identify limitations to our work. First, with regard to our review of the literature, the limitations are also the cases with few patients and the review based solely on PubMed publications. One of the main biases of our article is the difficulty of producing a complete collection, probably with coding problems and completeness of files. We also considered the issue of a recruitment bias with overdiagnosis in the face of the five cases diagnosed in 2022. The necrosis diagnosis of an adrenal gland is a diagnosis by trained radiologists. We do not think the Nantes University Hospital made an overdiagnosis. However, radiologists were probably aware of the aspect of the adrenal glands because of this sequence of patients, with more particular attention paid to the reading of the imaging at adrenal glands level, in view of this kind of clinical picture. The diagnostic criteria used by radiologists have notably been described by Chagué et al. [7]. On a CT scan, the glands typically appeared enlarged, hypodense and non-enhanced. On MRI, hyperintense enlarged glands were described on T2 and diffusion sequences, without hyper intensity on T1. Remember that gadolinium injection is contraindicated during pregnancy, and that MRI should be favored over computed tomography, in order to limit fetal irradiation. Moreover, as Chasseloup F et al. [8] wrote, we believe it is rather a matter of underdiagnosis, in view of the ignorance of the pathology and the absence of consensual management in the literature.

## 5. Conclusions

Adrenal necrosis during pregnancy therefore seems to be a pathology of the third trimester in young women with no particular history and primiparous.

It is important to mention it when faced with an evocative clinical picture during pregnancy, namely sudden and intense abdominal pain, morphine-requiring with an isolated biological inflammatory syndrome. The diagnosis is based on imaging, CT or MRI, depending on access possibilities. Curative anticoagulation is then quickly instituted for a period of three to six months. It is necessary to ensure the absence of adrenal insufficiency, even if it remains unlikely in the event of unilateral involvement. No factor seems to predict or anticipate this event during pregnancy.

A thrombophilia assessment should be carried out systematically and mainly to search for antiphospholipids. Indeed, currently, there is no consensus concerning preventive anticoagulation in the event of the next pregnancy; the only recommendations that mention this pathology (American recommendations) tend to be against prevention. On the other hand, we believe that the presence of thrombophilia (and more particularly of acquired thrombophilia) would encourage us to recommend special precautions for subsequent pregnancies.

It would be interesting to go further by comparing our population with another outside of pregnancy to judge the thrombotic factor generated by pregnancy, the laterality of the glandular involvement and any risk factors.

## Figures and Tables

**Table 1:** General characteristics of the population and clinical and paraclinical diagnostic criteria.

Patients	A	B	C	D	E	F	G	H
Age (years)	22	33	30	18	30	32	30	23
Parity	1	2	0	0	0	1 (September 2021)	0	1
BMI (kg/m^2^)	21.8	31.6	22.5	27	42	26.5	36.7	22.2
Term at diagnosis (weeks)	39	36	34	32	37	35	39	35
Year of diagnosis	October 2015	June 2018	August 2018	April 2022	August 2022	October 2022	October 2022	November 2022
Clinical								
Abdominal pain	Yes	Yes	Yes	Yes	Yes	No	Yes	Yes
Location	Right hypochondrium Transfixing irradiation Basithoracic	Right hypochondrium	NK	Right hypochondrium Brutal Lumbar radiation Murphy +	Right hypochondrium Transfixing Right flank radiation	Lumbar then brutal bilateral dorsolumbar	Sudden right basithoracic and right hypochondrium	Lumbar and right flank Dorsal radiation
Initial diagnosis evoked	NK	Stone pathology, renal colic	NK	NK	NK	Pre-eclampsia with incomplete picture, adrenal vein thrombosis, aortic dissection	Aortic dissection	NK
Imaging								
Ultrasound	Abdominal: normal	Abdominal: normal	Doppler of normal MI	Abdominal: normal Blade of left pleural effusion.	Hepatomegaly steatotic	Abdominal: normal Doppler of normal MI	0	Abdominal: normal
Injected CT	Adrenal necrosis Right adrenal vein thrombosis	Adrenal necrosis Hypertrophic and infiltrated aspect of the right adrenal gland	Right hemorrhagic necrosis Hemorrhagic edematous aspect of the right adrenal gland No IVC thrombus	Right adrenal vein thrombosis extended in the retrohepatic IVC	Right hemorrhagic necrosis	Bilateral adrenal necrosis Necrosis of the adrenals in a bilateral way.	Right adrenal necrosis Enlarged right gland Necrosis vein	Right adrenal necrosis Right adrenal necrosis.
NHAI/HAI	NHAI	NHAI	HAI	NHAI	HAI	NHAI	NHAI	NHAI
Diagnostic delay (hours)	11	NK	NK	>48	12	11	1	25

NHAI: nonhemorrhagic adrenal infarction; HAI: hemorrhagic adrenal infarction; NK: not known.

**Table 2:** Summary of the literature review.

24 Cases	
Maternal age	Mean 27.8 years, σ = 5.2 (19–38)
Term	Mean 29.8 SA, σ = 6.04 (16–38)
Parity (*n* = 19)	Mean 1.1, σ = 1.82 (0–7)
Background	0
Side	14 damages to the right gland 6 damages to the left gland 2 bilateral attacks from the outset 2 successive bilateral attacks on the right then on the left
HAI or NHAI	23 NHAIs (95.8%) 1 HAI
Adrenal insufficiency	2: - Left: Hydrocortisone supplementation - Bilateral: supplementation with hydrocortisone and fludrocortisone
Thrombophilia assessment (*n* = 23)	14 neg (60.8%) 3 MTHFR 677 CC gene mutations (including 1 increased factor VIII activity) 3 proteins S lowered (including 2 increased factor VIII activity) 1 circulating lupus anticoagulant 1 Factor V Leiden mutation1 increased factor VIII activity
Treatment	- Anticoagulation (curative or preventive, not specified) - Duration: from during pregnancy until 11 months postpartum No anticoagulation treatment n = 5
Follow up (*n* = 19)	**Imaging**: no consensus (*n* = 11) - CT or MRI - Lead time from 1 to 7 months - Normal or atrophic gland - No follow-up (*n* = 9) - 1 MRI at 3 years for suspected PE: normal gland

**Table 3:** Comparison of cases from the Nantes University Hospital with cases found in the literature.

32 Cases	Nantes University Hospital (*n* = 8)	Literature (*n* = 24)
Maternal age (years)	27.25	27.8
Term (weeks)	35.9	29.8
Parity	0.625	1.1
Side	7 right, 1 bilateral	14 right, 6 left, 4 bilateral
HAI or NHAI	6 NHAIs, 2 HAIs	23 NHAIs, 1 HAI
Adrenal insufficiency	0	2
Thrombophilia assessment	6 negatives 2 antiprothrombinase antibodies 1 antiprothrombinase antibody at 6 months	14 negatives
Treatment	Curative anticoagulation for 3 to 6 months	Anticoagulation, variable duration
Follow up	CT at 3 months: normal or atrophic gland	Imaging at 1–7 months: normal or atrophic gland

## Data Availability

Data of the case report are available on request in the Hospital files and were found thanks to the Nantes University Hospital Data Clinic’s epidemiologist. No new data were created for the systematic review.
